# Higher Risk Perception of HIV than of Chlamydia and HPV among Secondary School Students in Two German Cities

**DOI:** 10.1371/journal.pone.0061636

**Published:** 2013-04-24

**Authors:** Florence Samkange-Zeeb, Saskia Pöttgen, Hajo Zeeb

**Affiliations:** Leibniz Institute for Prevention Research and Epidemiology, Bremen, Germany; IPO, Inst Port Oncology, Portugal

## Abstract

**Background:**

Chlamydia and genital human papillomavirus (HPV) are the two most common sexually transmitted infections (STIs) among teens and young adults in industrialised countries. The majority of adolescents, however, have limited or no knowledge of these infections. Within the context of a cross-sectional survey on awareness and knowledge of sexually transmitted infections, secondary school students attending the 8th grade and above in Bremen and Bremerhaven, two cities in northern Germany, were asked to rate the risk of peers to get infected with HIV, HPV or chlamydia.

**Methods:**

Between October and December 2011, students aged 12–20 years completed an anonymous, self-administered questionnaire at their school. In addition to answering questions on awareness and knowledge of sexually transmitted infections, all students were also asked to rate the risk of peers to get infected with HIV, HPV or chlamydia. Furthermore, those reporting ever having sexual intercourse were asked to rate their own risk of getting infected with each of the three infections.

**Results:**

1,148 students, 55% female, completed the questionnaire. 27% of the students reported having had sexual intercourse. 68% of all students rated the risk of same-aged students to get infected with HIV/AIDS as high/medium. The corresponding proportions for HPV and chlamydia were 19 and 25% respectively. Those reporting ever having sexual intercourse generally perceived their own risk of getting infected with HIV, chlamydia or HPV as lower than that of their peers.

**Conclusion:**

Generally, the risk of getting infected with HIV was perceived as being higher than that of getting infected with HPV or chlamydia, most likely due to the fact that the students were more aware of HIV than of the other two infections. Efforts should be made to improve awareness and knowledge of HPV and chlamydia among school going adolescents, and to make them realize that these are common infections that are preventable.

## Introduction

The average age of first sexual intercourse in western European countries is reported to be decreasing [1] and adolescents are said not to use condoms consequently [2]–[4], placing them at risk of unplanned pregnancies and sexually transmitted infections (STIs). According to recent data, an increase in STIs such as chlamydia, syphilis and gonorrhea has been observed in several western European countries, particularly among young people aged 16–19 years [1], [5]–[9]. Although the HIV epidemic is reported to be generally stable in this region, increased rates of transmission have been observed in a number of countries, especially among men who have sex with men [10]. Heterosexual intercourse is however currently presumed to be the most common mode of transmission [11]. Thus, there is a non-negligible risk of adolescents to be infected with a sexually transmitted infection if they do not take preventive measures [12].

Chlamydia and genital human papillomavirus (HPV) are the two most common sexually transmitted infections (STIs) among teens and young adults in industrialised countries, affecting mostly females [13]. Similar to HIV, both infections can be asymptomatic; hence most people are not aware that they are infected. If not treated, chlamydia can lead to pelvic inflammatory diseases in women and painful infection in men, and possibly to reduced fertility or infertility in both sexes. Some HPV types lead to genital warts, while others can lead to cancer of the cervix, penis, anus or oropharynx [14]–[17]. Although both diseases are more prevalent than HIV/AIDS among adolescents in industrialised countries, awareness of these STIs in this population group is lower than that of HIV/AIDS [18], [19]. Awareness and knowledge of a risk, such as an STI, is closely linked to risk perception, and, together with other influencing factors such as perceived severity, benefits or barriers, assumed to shape health behavior [20]. To be able to perceive the personal risk for a particular STI, one needs to be equipped with basic information on the infection, such as how prevalent it is, how it presents itself, how one can be infected, and where one can go for testing or for help [21]. Awareness of a potential risk leads to it being better perceived, and to more concern being shown [22]. According to the Health belief Model, a high perception of risk leads to reduced risk taking and/or encourages protective health behavior [23]. This assumption, however, has been challenged by studies that find only a weak positive or even a negative association between perceived risk and subsequent preventive behavior [24], [25]. Likewise, the effect of knowledge and awareness on changing attitudes and behaviour appears limited [26]–[29]. Nevertheless, the three aspects, awareness, knowledge and risk perception, which are interlinked, are crucial components of sex education which help promote informed, healthy choices [30]–[32].

We assessed the awareness and knowledge of sexually transmitted infections among secondary school students attending the 8^th^ grade and above in Bremen and Bremerhaven, two cities in northern Germany, using an anonymous questionnaire. The students were also asked to rate the risk of peers to get infected with HIV, HPV and chlamydia. Those reporting ever having sexual intercourse were asked to rate their own risk of getting infected with each of the three named infections. The results of the analyses on awareness and knowledge have been published elsewhere [33]. In this paper we focus on STI risk perception. We were interested in assessing the adolescents' appraisal of the risk of peers to get infected with an STI, as well as the appraisal of those already sexually active of their own risk to get infected with an STI.

## Methods

The study was conducted in 8 secondary schools in two cities in northern Germany, Bremen and Bremerhaven, among 12–20 year old students attending the 8^th^ grade and above. Approval to conduct the study was received from the ethics commission of the University of Bremen, the Senator for Education, Science and Health and the Data Protection Officer for the State of Bremen.

As described in our manuscript on knowledge and awareness of STIs [33], the principals of 18 randomly selected secondary schools in Bremen and Bremerhaven were contacted and requested for permission to conduct the survey in their school. Each school was sent a flyer with information about the survey, including details on issues concerning informed consent and data protection. Eight schools, 6 in Bremen and 2 in Bremerhaven, agreed to cooperate with us. Both school types offered in the study region were included: those offering up to the 10^th^ grade only, and those offering up to the 12^th^ or 13^th^ grade.

The teachers, parents and students were provided with written information on the study. The study information was delivered to each school and the class teachers distributed the information to the students, who in turn passed on the study information, including consent forms, to their parents. In all the 8 participating schools, study information was provided for each student attending the 8^th^ grade and above. There was no selection of individual classes or particular grades. Information for parents, including consent forms, was also provided in Turkish and Russian.

Only students with signed parental or own consent (for those aged 18 and above) could take part in the survey.

### The questionnaire

On the day of the survey students with signed consent completed an anonymous, self-administered questionnaire at their school during normal school time. In the first section of the questionnaire socio-demographic information such as age, country of birth and parents' school education were collected. The second section covered issues on knowledge and awareness of STIs.

Questions on knowledge and awareness of STIs and on risk perception were constructed based on questionnaires used in other studies [34]–[38]. The questionnaires (one for girls and one for boys) were pre-tested on a sample of school-going adolescents aged 13–15 years. The pre-test participants were recruited from a school in Bielefeld, a city about 180 km from Bremen. The completed questionnaires were assessed for ambiguity, clarity, comprehensibility, and completion times required, and were then modified accordingly.

The students were asked which of the following infections, listed in the questionnaire, they had ever heard of: HIV/AIDS, human papillomavirus (HPV), chlamydia, herpes, syphilis, gonorrhoea and hepatitis B. This was followed by several questions on awareness and knowledge of STIs, covering specific issues on HPV awareness and general knowledge on sexually transmitted infections. The latter included questions on whether the students knew that using a condom protects against getting an STI, that infections such as HIV and HPV are treatable, but not curable, and that there is no vaccine against HIV/AIDS and chlamydia. Thereafter, the students were asked to rate the risk of same-aged adolescents to get infected with HIV/AIDS, HPV or chlamydia. The following response possibilities were offered: high, medium, low or don't know. In the last section students were asked if they had ever had sex, and if yes, their age at sexual debut, and the method of contraception they had used then, and the method they used now. Those reporting ever having sex were asked to rate their own risk of getting infected with HIV/AIDS, HPV or chlamydia. Again the following response possibilities were offered: high, medium, low or don't know.

### Analysis

Data analyses were conducted using SAS version 9.2.

#### Variables included

We used maternal educational status as an indicator of socioeconomic status. The variable father's education was excluded from the analyses as there were many missing values. Responses to the outcome variables peer and own risk perception for HIV, HPV and chlamydia were categorised as ‘high/medium’, ‘low’ and ‘don't know’. Students who did not responded to the questions on rating were excluded from all analyses.

#### Statistical methods

We performed descriptive statistics and calculated frequencies for all variables. Chi-squared tests were used to assess bivariate relations between the independent variables age, gender, migrant background, school education of mother, ever had sex, and type of school and the outcome variables peer and own risk perception for HIV, HPV and for chlamydia. For those reporting ever having sex, additional tests for use of condoms at sexual debut and currently and a comparison between indicated peer risk perception and own risk perception were done. As the outcome variables were polytomous, multivariable analyses were conducted using standard ordinal regression analysis (PROC LOGISTIC) with backward selection, including all independent variables. Ordinal logistic regression assumes that the relationship between each pair of outcome groups is the same. In our case this would mean that the coefficients describing the relationship between the category ‘don't know’ versus the category low is the same as that which describes the relationship between the category ‘low’ and the category ‘medium/high’. To assess the appropriateness of the ordinal regression model, the proportional odds assumption was tested and the score test (chi square) was not significant.

## Results

The study population and the findings on knowledge and awareness of sexually transmitted infections have already been reported [33]. Here the study population will be briefly described, as will observations regarding STIs adolescents had heard of.

1148 students, (632 girls and 516 boys), aged 12–20 years, participated in the study (response rate 28%). 31% of the students had a migrant background (i.e. they themselves, or one/both of their parents were born abroad) and 27% reported ever having sexual intercourse. The average age of first sex was 15 years and the average number of lifetime sexual partners was two. Among those who reported ever having sex, 81% (76% of the girls and 86% of the boys) reported using a condom at sexual debut and 65% (79% of the girls and 51% of the boys) reported currently using condoms.

Almost all students (99%) had heard of HIV/AIDS, 91% of herpes, 83% of hepatitis, 23% of chlamydia, 17% of gonorrhea and 13% of HPV. Gender differences were observed for HPV, chlamydia, syphilis and gonorrhea, with significantly more girls than boys reporting having heard of HPV and chlamydia, and significantly more boys than girls reporting having heard of syphilis and gonorrhea.

### Risk perception for peers

Of the three infections for which the students were asked to rate the risk of getting infected for same-aged adolescents, HIV/AIDS had the least proportion of students who responded with “don't know” or with missing responses (4%). The corresponding proportions for HPV and chlamydia were 65% and 60%, respectively. Whereas 68% of the students rated the risk of same-aged students to get infected with HIV/AIDS as high/medium, the corresponding figures where 19% for HPV and 25% for chlamydia. In bivariate analyses, gender was significantly associated with risk perception for all three infections, with a higher proportion of girls rating the risk of getting infected as high/medium (table 1). In comparison to students whose mothers had a low/medium school education, a higher proportion of students whose mothers had a high level school education rated the risk of peer to get infected with HIV/AIDS as low. For HPV and chlamydia, no differences based on maternal school education were observed. In comparison to those reporting no sexual experience, a higher proportion of students who reported ever having sex rated the risk of peers to get infected with HPV and chlamydia as high/medium. In multivariable analyses, age and gender were significant predictors for HIV risk perception for peers, with younger students and females more often rating the risk of getting infected as high/medium. For HPV risk perception, only the variable ever having sex remained a significant predictor. For chlamydia risk perception, age and ever having sex remained significant predictors (table 2).

**Table 1:** Results of bivariate analyses for STI risk perception for peers and various predictor variables.

	HIV risk perception for peers				HPV Risk perception for peers				Chlamydia risk perception for peers			
	High/med (n/%)	Low (n/%)	*DK (n/%)	**p value	High/med (n/%)	Low (n/%)	*DK (n/%)	**p value	High/med (n/%)	Low (n/%)	*DK (n/%)	**p value
Age in years												
≤14	366 (71)	131 (25)	18 (4)	0.09	84 (17)	74 (15)	341 (68)	**<0.01**	98 (20)	63 (13)	339 (68)	**<0.01**
≥15	414 (67)	191 (31)	15 (2)		136 (23)	109 (18)	277 (59)		187 (31)	114 (19)	246 (50)	
Gender												
Female	474 (76)	135 (22)	13 (2)	**<0.01**	138 (23)	85 (14)	382 (63)	**<0.01**	176 (29)	76 (13)	356 (59)	**<0.01**
Male	306 (60)	187 (36)	20 (4)		82 (17)	98 (20)	314 (64)		109 (22)	101 (20)	287 (58)	
School education of mother												
High	255 (63)	135 (34)	12 (3)		71 (18)	63 (16)	254 (65)		101 (26)	58 (15)	230 (59)	
Middle	236 (74)	79 (25)	3 (1)	**<0.01**	72 (24)	56 (18)	178 (58)	0.14	70 (23)	63 (21)	172 (56)	0.12
Low	70 (66)	33 (31)	3 (3)		24 (24)	20 (20)	57 (56)		27 (26)	19 (19)	57 (55)	
Unknown	219 (71)	75 (5)	15 (5)		53 (17)	44 (14)	207 (68)		87 (28)	37 (12)	184 (60)	
Migrant background												
no	523 (68)	229 (30)	18 (2)	0.15	138 (18)	127 (17)	482 (65)	0.14	190 (25)	128 (17)	434 (58)	0.40
yes	246 (71)	87 (25)	13 (4)		79 (36)	54 (16)	200 (60)		88 (26)	46 (14)	200 (60)	
Ever had sex												
No	560 (69)	224 (28)	27 (3)	0.36	139 (18)	124 (16)	525 (67)	**<0.01**	166 (21)	118 (15)	507 (64)	**<0.01**
yes	213 (68)	94 (30)	6 (2)		79 (26)	56 (19)	166 (55)		117 (39)	56 (18)	131 (43)	
School type												
up to 10^th^ grade	244 (68)	105 (29)	12 (3)	0.77	64 (18)	64 (18)	221 (63)	0.45	86 (25)	57 (16)	205 (59)	0.85
Up to 12^th^/13^th^ grade	536 (69)	217 (28)	21 (3)		156 (21)	119 (16)	475 (63)		199 (26)	120 (16)	438 (58)	

* DK = Don't know.

** p-value Chi-square.

**Table 2:** Results of multivariable regression analyses for STI risk perception for peers^a^.

	HIV risk perception for peers	HPV Risk perception for peers	Chlamydia risk perception for peers
	OR: 95% CI	OR: 95% CI	OR: 95% CI
Age in years			
≥15 (ref)	1.00	1.00	1.00
≤14	1.38: 1.02–1.85*	0.77: 0.58–1.02	0.59: 0.45–0.78*
Gender			
Male (ref)	1.00	1.00	1.00
Female	2.07: 1.59–2.68*	1.10: 0.86–1.41	1.13: 0.88–1.44
School education of mother			
Low (ref)	1.00	1.00	1.00
Middle	1.38: 0.85–2.26	0.91: 0.58–1.42	0.94: 0.60–1.48
High	0.85: 0.53–1.36	0.69: 0.44–1.08	0.96: 0.61–1.49
Unknown	1.24: 0.76–2.02	0.57: 0.38–0.90	0.88: 0.56–0.98
Migrant background			
yes (ref)	1.00	1.00	1.00
no	0.90: 0.66–1.20	0.81: 0.62–1.06	1.03: 0.79–1.35
Ever had sex			
no (ref)	1.00	1.00	1.00
yes	1.12: 0.81–1.55*	1.59: 1.17–2.15*	2.01: 1.29–2.70*
School type			
up to 10^th^ grade (ref)	1.00	1.00	1.00
Up to 12^th^/13^th^ grade	1.10: 0.82–1.48	0.89: 0.67–1.18	0.74: 0.57–0.98

a ordinal regression models, all scores range from 0 to 2, odds ratios indicate effects per one point difference.

* statistically significant odds ratios.

ref = reference group.

### Own risk perception

Of the 314 students who reported ever having sex, 5% responded ‘don't know’ to the question on own HIV risk perception. The corresponding proportions for HPV and chlamydia were 34% and 29%, respectively. Among those who rated their own risk of getting infected with HIV, 16% perceived their risk as high/medium (HPV 9%, chlamydia 13%). Results of bivariate analyses showed an association between age and own risk perception for HIV and chlamydia, with a higher proportion of students older than 14 years reporting their risk of getting infected as low. Students younger than 14 years and those with a migrant background more often responded to the question on HIV risk perception with ‘don't know’ (table 3). We did not observe any significant predictors in multivariable analyses.

**Table 3:** Results of bivariate analyses for own risk perception among those reporting having had sexual intercourse (fisher's exact test).

	HIV risk perception for peers				HPV Risk perception for peers				Chlamydia risk perception for peers			
	High/med (n/%)	Low (n/%)	*DK (n/%)	**p value	High/med (n/%)	Low (n/%)	*DK (n/%)	**p value	High/med (n/%)	Low (n/%)	*DK (n/%)	**p value
Age in years												
≤14	6 (22)	18 (67)	3 (11)	0.01#	3 (11)	11 (41)	13 (48)	0.22	4 (15)	10 (37)	13 (48)	0.05#
≥15	43 (15)	228 (81)	12 (4)		24 (9)	162 (58)	93 (33)		34 (12)	169 (60)	77 (28)	
Gender												
Female	22 (14)	135 (84)	4 (2)	0.06	16 (10)	90 (57)	52 (33)	0.63	22 (14)	95 (60)	42 (26)	0.47
Male	27 (18)	111 (75)	11 (7)		11 (7)	83 (56)	54 (36)		16 (11)	84 (57)	48 (32)	
School education of mother												
High	18 (20)	68 (77)	2 (2)		11 (13)	49 (56)	27 (31)		13 (15)	50 (58)	23 (27)	0.68
Middle	12 (15)	64 (80)	4 (5)	0.39	5 (6)	50 (63)	25 (31)	0.63	9 (11)	48 (60)	23 (29)	
Low	4 (12)	25 (76)	4 (12)		3 (9)	16 (50)	13 (40)		4 (12)	15 (45)	14 (42)	
Unknown	15 (14)	89 (82)	5 (5)		8 (7)	58 (54)	41 (38)		12 (11)	66 (61)	30 (28)	
Migrant background												
no	33 (15)	183 (82)	6 (3)	0.02#	19 (9)	125 (57)	75 (34)	0.90	28 (13)	131 (60)	61 (28)	0.57
yes	15 (19)	58 (72)	8 (10)		8 (10)	44 (55)	28 (35)		10 (13)	43 (54)	27 (34)	
School type												
up to 10^th^ grade	6 (14)	35 (81)	2 (5)	0.95	4 (9)	22 (51)	17 (40)	0.70	3 (7)	24 (56)	16 (37)	0.33
Up to 12^th^/13^th^ grade	43 (16)	211 (79)	13 (5)		23 (9)	151 (57)	89 (34)		35 (13)	155 (59)	74 (28)	
Condom used at sexual debut												
no	9 (16)	45 (78)	4 (7)	0.65	3 (5)	32 (56)	22 (39)	0.55	6 (10)	33 (57)	19 (33)	0.76
yes	40 (16)	201 (80)	11 (4)		24 (9)	141 (57)	84 (34)		32 (13)	146 (59)	71 (29)	
Condom currently used												
no	14 (13)	92 (84)	3 (3)	0.23	8 (7)	64 (59)	36 (33)	0.71	14 (13)	65 (60)	30 (28)	0.88
yes	35 (18)	153 (77)	12 (6)		19 (10)	108 (55)	70 (36)		24 (12)	113 (57)	60 (30)	

* DK = Don't know.

** p- value - fisher exact test.

# statistically significant p-values.

### Comparison between peer and own risk perception

For HIV, 27% of the students reporting ever having sexual intercourse perceived their own risk of getting infected and that of their peers as low, while 51% perceived their own risk as low, but that of their peers as high/medium (table 4). For HPV, 15% of the students reported their own risk and that of peers as low, and 17% reported low own risk but high/medium peer risk. For chlamydia, 15% of the students perceived their own risk of getting infected and that of their peers as low, while 25% perceived their own risk to be low but that of their peers as high/medium. The differences in own and peer risk perception for all three infections were statistically significant.

**Table 4:** Comparison of peer and own risk perception rating for HIV/AIDS, HPV and chlamydia infection among students reporting having had sexual intercourse.

	Own risk perception			*p-value
Peer risk perception	High/medium	low	Don't know	
HIV/AIDS (n = 309)				
High/medium	14%	51%	3%	
Low	2%	27%	1%	<0.01
Don't’ know	0%	1%	1%	
HPV (n = 299)				
High/medium	6%	17%	4%	
Low	2%	15%	2%	<0.01
Don't know	1%	25%	29%	
Chlamydia (n = 301)				
High/medium	10%	25%	3%	
Low	2%	15%	2%	<0.01
Don't know	1%	18%	24%	

* p- value - fisher exact test.

## Discussion

In the context of a cross-sectional survey on awareness and knowledge of sexually transmitted infections, secondary school students attending the 8^th^ grade and above were asked to rate the risk of peers to get infected with HIV, HPV and chlamydia. Those reported ever having sexual intercourse were also asked to rate their own risk of getting infected with the three infections. Generally, the students were able to rate the risk of peers to get infected with HIV, with only a small proportion responding with ‘don't’ know’. In contrast, the majority of students responded with ‘don't know’ for chlamydia and HPV. Similarly, the lowest proportion of ‘don't know’ for own risk perception was observed for HIV. All in all, students rated their peers to be at higher risk of getting infected with HIV than with chlamydia or HPV. Those reporting ever having sex generally rated their own risk of getting infected with each of the three infections to be lower than that of their peers.

There is a lack of epidemiological data on risk perception of infection with sexually transmitted infections among school-going adolescents in Germany and our study helps fill this gap. Results of a number of surveys on awareness and knowledge of HIV/AIDS, chlamydia and HPV among this population group have recently been published [18], [39]–[42].

As we conducted an observational cross-sectional study, our analyses were of an explorative nature. The estimated p-values can therefore only be regarded as indicative of a relationship between the independent variables and risk perception.

Although the low participation rate observed in our study can be partly explained by the sensitive nature of the research question and the reluctance of parents to allow their children to take part in such surveys, it still remains a limiting factor.

Our observation that only a small proportion of students responded to the question on risk perception for HIV with ‘don't know’, while the corresponding proportions for chlamydia and HPV are very high, is a further indication that adolescents are more aware of HIV than of the latter infections. As has been noted for Germany [18], [43], the emphasis of STI prevention programs in some industrialised countries is on HIV/AIDS. Consequently, young people perceive their own risk, or that of peers, to get infected with HIV to be higher than that to get infected with chlamydia or HPV, although the latter are more prevalent in these countries, especially among this population group [34], [44], [45].

In bivariate analyses we observed gender differences in risk perception for peers for all three infections, with girls more often than boys perceiving the risk of getting infected to be high/medium. Interestingly, we did not make the same observation for own risk perception. Here the majority of students, irrespective of gender, perceived their own risk of getting infected as low. We also did not observe any statistically significant differences based on use of condoms at sexual debut or currently. The low perception of own risk, particularly for chlamydia and HPV, can be taken as a further indication of the lack of awareness of these infections among the study population. Findings of a chlamydia prevalence study conducted among sexually active school girls aged 14–17 years conducted in 2004 in Berlin, Germany, serve to underline this assumption. In that study, 83% of the participants had never heard of chlamydia. 6% of the participants were found to be infected with chlamydia, with the highest prevalence (10%) being observed among those 17 years old [43].

A comparison of the perceived risk for peers and own perceived risk showed that the students, irrespective of gender, generally perceived their own risk to get infected as lower than that of their peers. This discrepancy in risk perception was particularly observed for HIV, the STI almost all students had heard of, with 51% of the students perceiving their own risk of infection to be low, but that of their peers to be high/medium. For HPV and chlamydia, the STIs students were less aware of, the discrepancy was less marked. According to literature, individuals generally perceive themselves to be less at risk than others, particularly where the risk is controllable or behavior related [46], [47], a phenomenon known as ‘optimistic bias’. This observation has also been made among adolescents, who during the change from childhood to adulthood believe that negative effects of risky behavior only happen to others [48].

The concept of risk perception is based on the understanding that an individual is more likely to take preventive measures when he or she believes to be personally at risk [23]. Although this aspect is debatable, as is the case with the effect of awareness and knowledge on behaviour change [26]–[29] it is a core component of public health interventions aiming at encouraging the adoption of healthier behavior [49]. For HIV, the intensive prevention and information campaigns, particularly in industrialised countries, led to high awareness and knowledge of the infection, and to a high risk perception. This in turn probably led to the infection not spreading the way it could have otherwise done, with ensuing low factual risk for the broad population. The dreaded aspect of HIV/AIDS also played and continues to play a role, while other STIs do not have the same dreaded potential. Awareness and knowledge are important prerequisites for risk perception, as it is not possible to perceive the risk of something one is not aware of or has no knowledge of [22]. Given this basic fact, remodeling of STI prevention curricula in schools should be considered to support a more realistic and differentiated risk perception on STIs among adolescents, including a focus on preventability.

## References

[1] Adler MW (2006) Sexually transmitted infections in Europe. Eurohealth 12: 3–6.

[2] Ford N (1992) The AIDS awareness and sexual behaviour of young people in the South-west of England. J Adolesc 15: 393–413. 1487576 10.1016/0140-1971(92)90071-c

[3] Editorial team (2005) Young people's knowledge of sexually transmitted infections and condom use surveyed in England. Euro Surveill 10: 10 ((31)) pii = 2766. 16785677

[4] Warner LM, Hohmann C, Böhmer-Lasthaus S, Luszczynska A, Piko BF, et al (2007) Eine 4-Länder-Studie über sexuelles Schutzverhalten bei Jugendlichen. Zeitschrift für Gesundheitspsychologie 15 ((3)) 109–118.

[5] Panchaud C, Singh S, Feivelson D, Darroch JE (2000) Sexually transmitted diseases among adolescents in developed countries. Fam Plan Persp 32: 24–32 & 45. 10710703

[6] Berglund T, Fredlund H, Giesecke J (2001) Epidemiology of the re-emergence of gonorrhoea in Sweden. Sex Transm Dis 111–114. 11234784 10.1097/00007435-200102000-00009

[7] Nicoll A, Hamers FF (2002) Are trends in HIV, gonorrhoea and syphilis worsening in Western Europe? BMJ 324: 1324–1327. 12039830 10.1136/bmj.324.7349.1324 PMC1123279

[8] Twisselmann B (2002) Rising trends of HIV, gonorrhoea, and syphilis in Europe make case for introducing European surveillance systems. Euro Surveill 6 ((23)) pii = 1952 http://www.eurosurveillance.org/ViewArticle.aspx?ArticleId=1952. Accessed 2011 Nov 30.

[9] Health protection Surveillance Centre (2005) Surveillance of STI. A report by the Sexually Transmitted Infections subcommittee for the Scientific Advisory committee of the health Protection Surveillance Centre, http://www.hpsc.ie/hpsc/AboutHPSC/ScientificCommittees/Publications/File,1437,en.pdf. Accessed 2012 Oct 15.2

[10] AVERT HIV and AIDS in Western and Central Europe. Available http://www.avert.org/aids-europe.htm. Accessed 2012 Dec 11.

[11] UNAIDS (2008) North America, Western and Central Europe AIDS epidemic update: Regional summary http://data.unaids.org/pub/Report/2008/jc1532_epibriefs_namerica_europe_en.pdf Accessed 2013 Jan 02.

[12] Rosengard C, Adler NE, Millstein SG, Gurvey JE, Ellen JM (2004) Perceived STD risk, relationship, and health values in adolescents' delaying sexual intercourse with new partners. Sex Transm Infect 80: 130–137 doi: 10.1136/sti.2003.006056. 15054176 10.1136/sti.2003.006056 PMC1351212

[13] Centers for Disease Control and Prevention (CDC) (2012) Sexually Transmitted Diseases (STDs). Available: http://www.cdc.gov/std/hpv/stdfact-hpv.htm. Accessed 2012 Dec 12.

[14] MacDonald NE, Brunham R (1997) The Effects of Undetected and Untreated Sexually Transmitted Diseases: Pelvic Inflammatory Disease and Ectopic Pregnancy in Canada. The Canadian Journal of Human Sexuality 6 ((2)) Special Issue: STDs and Sexual/Reproductive Health.

[15] Department of Health (DH) (2012) Human papillomavirus. The Green Book. Chapter 18a, pp. 1–2. Avaialble: https://www.wp.dh.gov.uk/…/07/Green-Book-Chapter-18a-v2_0.pdf. Accessed 2012 Dec 12

[16] Simms I, Stephenson JM (2000) Pelvic inflammatory disease epidemiology; What do we know and what do we need to know? Sex Trans Inf 76: 80–87. 10.1136/sti.76.2.80 PMC1758284 10858707

[17] Forster AS, Marlow LAV, Wardle J, Stephenson J, Waller J (2012) Interest in having HPV vaccination among adolescent boys in England. Vaccine 30: 4505–4510. 22561487 10.1016/j.vaccine.2012.04.066

[18] Lengen C, Jäger S, Kistemann T (2010) The knowledge, education and behaviour of young people with regard to Chlamydia trachomatis in Aarhus, Denmark and Bonn, Germany: do prevention concepts matter? Soc Sci Med 70 ((11)): 1789–98. 20307923 10.1016/j.socscimed.2010.01.048

[19] Samkange-Zeeb F, Spallek L, Zeeb H (2011) Knowledge and awareness of sexually transmitted diseases among European adolescents: a systematic review. BMC Public Health 11: 727. 21943100 10.1186/1471-2458-11-727 PMC3189891

[20] Leval A, Sundström K, Ploner A, Dahlström LA, Widmark C, et al.(2011) Assessing perceived risk and STI prevention behaviour: A national population-based study with special reference to HPV. PLoS ONE 6:6 Available: http://www.plosone.org/article/info%3Adoi%2F10.1371%2Fjournal.pone.0020624. Accessed 2012 Sept 15. 10.1371/journal.pone.0020624 PMC3107227 21674050

[21] Gilbert S (2011) Getting more young women screened for chlamydia: findings from qualitative research. National Chlamydia Coalition 3. Available: http://www.prevent.org/data/images/ncc/ncc%20research%20brief%203.pdf. Accessed 2012 Oct 15.

[22] BVSDE - Virtual Library in Sustainable Development and Environmental Health Risk perception. Available: http://www.bvsde.paho.org/tutorial6/i/pdf/topic_04.pdf. Accessed 2012 Oct 15.

[23] Hayden JA (2009) Health Belief Model. In: Introduction to Health Behavior Theory. Jones and Bartlett Publishers. pp. 31–36. Available: http://www.jblearning.com/samples/0763743836/chapter%204.pdf. Accessed 2012 Oct 15.

[24] Gerard M, Gibbons FX, Bushman BJ (1996) Does perceived vulnerability to HIV motivate precautionary sexual behaviour? A critical review of the literature. Psychological Bulletin 119: 390–409. 8668745 10.1037/0033-2909.119.3.390

[25] van der Plight J (1998) Perceived risk and vulnerability as predictors of precautionary behaviour. British Journal of Health Psychology 2: 1–4.

[26] Lister-Sharpe D, Chapman S, Stewart-Brown S, Sowden A (1999) Health promoting schools and health promotion in schools: two systematic reviews. Health Technol Assess 3 ((22)) 1–207. 10683593

[27] Wight D, Raab G, Henderson M, Abraham C, Buston K, et al (2002) Limits of teacher delivered sex education: interim behavioural outcomes from randomised trial. BMJ 324: 1–6. 12065268 10.1136/bmj.324.7351.1430 PMC115856

[28] Stephenson J, Strange V, Forrest S, Oakley A, Copas A, et al (2004) Pupil-led sex education in England (RIPPLE study): cluster randomised intervention trial. Lancet 364 ((9431)): 338–346. 15276393 10.1016/S0140-6736(04)16722-6

[29] Tucker JS, Fitzmaurice AE, Imamura M, Penfold S, Penney GC, et al (2006) The effect of the national demonstration project Healthy Respect on teenage sexual health behaviour. Eur J Public Health 17 ((1)) 33–41. 16601108 10.1093/eurpub/ckl044

[30] WHO Regional Office for Europe (2001) Who Regional Strategy on Sexual and Reproductive Health. www.euro.who.int/document/e74558.pdf. accessed 17.03.2011

[31] Bobrova N, Sergeev O, Grechukhina T, Kapiga S (2005) Social-cognitive predictors of consistent condom use among young people in Moscow. Perspect Sex Reprod Health 37 ((4)) 174–178. 16380362 10.1363/psrh.37.174.05

[32] Bundeszentrale für gesundheitliche Aufklärung (2006) Country papers on youth sexuality in Europe – Synopsis. BZgA

[33] Samkange-Zeeb F, Mikolajczyk RT, Zeeb H (2011) Awareness Awareness and knowledge of sexually transmitted diseases among secondary school students in two German cities. Journal of Community Health DOI 10.1007s1090001296144/s10900-012-9614-4. 10.1007/s10900-012-9614-4 23001541

[34] Garside R, Ayres R, Owen M, Pearson VAH, Roizen J (2001) ‘They never tell you about the consequences’: young people's awareness of sexually transmitted infections. Int J STD & AIDS 12: 582–588. 11516367 10.1258/0956462011923750

[35] Woodhall SC, Lehtinen M, Verho T, Huhtala H, Hokkanen M, et al (2007) Anticipated acceptance of HPV vaccination at the baseline of implementation: a survey of parental and adolescent knowledge and attitudes in Finland. J Adolesc Health 40: 466-. 17448408 10.1016/j.jadohealth.2007.01.005

[36] Gottvall M, Larsson M, Högkund AT, Tydén T (2009) High HPV vaccine acceptance despite low awareness among Swedish upper secondary school students. Eur J Contr Repr Health Care 14: 399–405. 10.3109/13625180903229605 19929642

[37] Das A, Madhwapathi V, Davies P, Brown G (2010) Knowledge and acceptability of the HPV vaccine by school children and their parents in Birmingham. Vaccine 28 ((6)): 1440–6. 20005317 10.1016/j.vaccine.2009.11.041

[38] Pelucchi C, Esposito S, Galeone C, Semino M, Sabatini C, et al (2010) Knowledge of human papillomavirus infection and its prevention among adolescents and parents in the greater Milan area, Northern Italy. BMC Public Health 10: 378. 20584324 10.1186/1471-2458-10-378 PMC2901377

[39] Sachsenweger M, Kundt G, Hauk G, Lafrenz M, Stoll R (2011) Knowledge of school pupils about the HIV/AIDS topic at selected schools in Mecklenburg-Pomerania: Results of a survey of school pupils. Gesundheitswesen 73 ((1)): e21–6 doi: 10.1055/s-0029-1246199. 20198565 10.1055/s-0029-1246199

[40] Samkange-Zeeb F, Spallek L, Klug SJ, Zeeb H (2011) HPV infection awareness and self-reported HPV vaccination coverage in female adolescent students in two German cities. J Community Health 37 ((6)): 1151–6 doi: 10.1007/s10900-012-9589-1. 10.1007/s10900-012-9589-1 22772842

[41] Blödt S, Holmberg C, Müller-Nordhorn J, Rieckmann N (2012) Human papillomavirus awareness, knowledge and vaccine acceptance: A survey among 18–25 year old male and female vocational school students in Berlin, Germany. Eur J Public Health 22 ((6)): 808–13 doi: 10.1093/eurpub/ckr188. 22199161 10.1093/eurpub/ckr188

[42] Stöcker P, Dehnert M, Schuster M, Wichmann O, Delereé Y (2012) Human papillomavirus vaccine uptake, knowledge and attitude among 10th grade students in Berlin, Germany, 2010. Hum Vaccin Immunother 9 ((1)): 1–9. 10.4161/hv.22192 PMC3667949 22995838

[43] Gille G, Klapp C, Diedrich K, Schäfer A, Moter A, et al (2005) Chlamydien, eine heimliche Epidemie unter Jugendlichen - Prävalenzbeobachtung bei jungen Mädchen in Berlin (2005) Dtsch. Ärztebl Heft 28–29: 2021–2025.

[44] Clark LR, Jackson M, Allen-Taylor L (2002) Adolescent knowledge about sexually transmitted diseases. Sex Transm Dis 29 ((8)): 436–43. 12172527 10.1097/00007435-200208000-00002

[45] Forhan SE, Gottlieb SL, Sternberg MR, Xu F, Datte SD, et al (2009) Prevalence of sexually transmitted infections among female adolescents aged 14 to 19 in the United States. Pediatrics 124: 1505–1512. 19933728 10.1542/peds.2009-0674

[46] Harris PR, Griffin DW, Murray S (2008) Testing the limits of optimistic bias: Event and person moderators in a multilevel framework. J Pers Socl Psychol 95: 1225–1237. 10.1037/a0013315 18954204

[47] Klein CTF, Helweg-Larsen M (2002) Perceived control and the optimistic bias: A meta-analytic review. Psychol Health 17: 437–446.

[48] Busen NH, Marcus MT, von Sternberg KL (2006) What African-American middle school youth report about risk-taking behaviours. J Pediat Health Care 20 ((6)): 393–400. 10.1016/j.pedhc.2006.03.003 17071370

[49] Brewer NT, Weinstein ND, Cuite CL, Herrington JE (2004) Risk perceptions and their relation to risk behavior. Ann Behav Med 27 ((2)) 125–130. 15026296 10.1207/s15324796abm2702_7

